# Biomarker-Guided Implant Maintenance (BGIM): A Narrative Review and Protocol Proposal

**DOI:** 10.3390/jcm15072496

**Published:** 2026-03-24

**Authors:** Tiziano Testori, Richard Lazzara, Renzo Guarnieri, Massimo Del Fabbro

**Affiliations:** 1 Department of Biomedical, Surgical and Dental Sciences, University of Milan, 20122 Milan, Italy; info@tizianotestori.com; 2 Department of Implantology and Oral Rehabilitation, Dental Clinic, IRCCS Ospedale Galeazzi-Sant’Ambrogio, 20157 Milan, Italy; 3 Department of Periodontics and Oral Medicine, School of Dentistry, University of Michigan, Ann Arbor, MI 48109, USA; 4 Department of Oral Medicine, Infection and Immunity, Harvard School of Dental Medicine, Boston, MA 01451, USA; 5 Private Implant Dentistry and Periodontal Practice, West Palm Beach, FL 33409, USA; rjlazzara@gmail.com; 6 Private Periodontal Implant Practice, 31100 Treviso, Italy; renzoguarnieri@gmail.com; 7 Department of Oral and Maxillofacial Sciences, Sapienza University of Rome, 00161 Rome, Italy; 8 Department of Prosthodontics and Implantology, Saveetha Institute of Medical and Technical Sciences, Saveetha Dental College and Hospitals, Saveetha University, Chennai 600077, Tamil Nadu, India; 9 Fondazione Ca’ Granda IRCCS Ospedale Maggiore Policlinico, 20122 Milan, Italy

**Keywords:** aMMP-8, biomarker, dental implants, epidemiology, implant maintenance, peri-implantitis, risk assessment

## Abstract

Dental implants are a popular clinical procedure for the rehabilitation of fully and partially ede ntulous patients. There is long-term evidence that implant-supported dental prostheses represent a predictable treatment for replacing missing teeth. However, several types of complications may arise, which can compromise implant treatment outcome. Peri-implant disease is a growing biological complication, consisting of a progressive loss of supporting bone, associated with microbial biofilm and clinical inflammation. It represents a concern for clinicians and patients, having a negative impact on quality of life. This narrative review aimed at summarize the current knowledge on etiology, epidemiology, risk factors, and pathogenesis of peri-implant disease. It also focused on the diagnostic potential of active matrix metalloproteinase-8 (aMMP-8) in peri-implant sulcular fluid for assessing the status of peri-implant tissues and the risk of developing peri-implantitis. A literature search was conducted in PubMed and Scopus databases using search terms like: peri-implantitis, peri-implant biomarkers, aMMP-8, implant maintenance, risk assessment. Clinical studies, systematic reviews, meta-analysis and consensus papers published up to June 2025 were considered. Finally, based on the main factors involved in the onset and progression of peri-implant disease, a new protocol was conceived for determining the optimal implant maintenance scheduling for individual patients. The Biomarker-Guided Implant Maintenance (BGIM) protocol considers a few key parameters, among which aMMP-8 level, and proposes three categories associated with different levels of risk for peri-implantitis. The higher the risk, the more frequently a patient should undergo professional maintenance, to prevent peri-implant disease, with potential favorable effects on implant longevity. The proposed BGIM protocol, that requires prospective validation, represents a structured and clinically applicable biomarker-driven framework for individualizing implant maintenance scheduling by integrating real-time chairside quantification of aMMP-8 with established patient-related risk factors.

## 1. Introduction

Peri-implantitis is a multifactorial biological complication involving inflammation of peri-implant tissues and progressive loss of bony support that may lead to implant failure [1]. The susceptibility to peri-implantitis may vary in the population according to multiple risk factors and risk indicators, but the main etiological factor primarily consists of microbial biofilm accumulation [2,3].

Although several risk assessment tools for peri-implant disease have been proposed, including the Implant Disease Risk Assessment (IDRA) [4] and the Peri-implantitis Risk Assessment (PiRA) [5], these tools are relatively complex and time-consuming and are not specifically designed for repeated chairside use during routine maintenance visits. Moreover, they do not incorporate dynamic biological information derived directly from peri-implant tissues. Therefore, there is a need for a simplified and biologically driven framework that can support clinicians in determining individualized maintenance schedules.

To address this gap the aims of our work were:

- to review and synthesize current evidence relevant to peri-implant disease features, diagnosis and monitoring;
- to propose a new protocol (Biomarker-Guided Implant Maintenance (BGIM)) conceived by integrating a rapid chairside biomarker test with established patient-related risk indicators.

The BGIM protocol represents a risk-stratification tool and a recall-interval decision aid, intended to complement conventional clinical assessment. It acts as a screening adjunct for early detection of inflammation, aiming to identify patients at higher risk who may benefit from more frequent recall appointments than low-risk patients.

## 2. Methods

This narrative review was based on a literature search conducted in PubMed/MEDLINE and Scopus databases using the following keywords and MeSH terms alone and combined using Boolean operators: “peri-implantitis”, “peri-implant biomarkers”, “aMMP-8”, “implant maintenance”, and “risk assessment”. Articles published up to June 2025 were considered. Eligible publications included clinical studies, systematic reviews, meta-analyses, and consensus papers reporting on peri-implant disease epidemiology, diagnostic biomarkers, and maintenance strategies. The terms (“peri-implantitis” OR “peri-implant disease”), and the MeSH term “Peri-Implantitis” [Mesh] yielded respectively 5442 and 2730 results on Pubmed, while the same searches provided 11,297 and 6206 results on Scopus. The search was then restricted according to the specific topic to address in this review (for example “Peri-Implantitis/epidemiology” [Mesh] (*n* = 212 results), “Peri-Implantitis/etiology” [Mesh] OR “Peri-Implantitis/pathology” [Mesh] OR “Peri-Implantitis/physiopathology” [Mesh] (*n* = 1210 results), “Peri-Implantitis/diagnosis” [Mesh] (*n* = 412 results), (“peri-implantitis” OR “peri-implant disease”) AND (“aMMP-8” OR “active matrix metalloproteinase-8”) (*n* = 25 results)). Two authors (M.D.F. and R.L.) selected the papers based on the relevance with the topic intended to describe.

## 3. Prevalence and Incidence of Peri-Implant Disease

Previous studies reported that the prevalence of peri-implantitis may range from 14 to 43% [6,7]. The reported prevalence of peri-implantitis varies widely, depending on different factors, including the population, the follow-up period or the inconsistent disease definitions among published studies, highlighting the absence of a unified research standard. The 2017 World Workshop on the Classification of Periodontal and Peri-Implant Diseases and Conditions aimed at solving this issue and proposed a new classification of periodontal and peri-implant diseases [8]. Updated epidemiological studies reported a prevalence of 19.5% and 12.5% at the patient level and at the implant level, respectively [9,10]. Remarkably, a cross-sectional study showed that application of the new diagnostic criteria considerably reduces the reported prevalence of peri-implantitis [11]. Further recent literature highlighted that the prevalence and incidence of peri-implantitis is still highly variable in the population being up to 45% at the patient level, and 22–23% at the implant level [9,12,13]. Astolfi et al. (2022) in a study of 555 patients reported a 16.9% of clinically identifiable peri-implantitis [14]. They also found the incidence in patients without maintenance had a much higher rate (61.4%) [14]. Similar results were previously reported in 2012 in a 5-year follow-up study by Oliveira Costa et al. (2012) who concluded that patients not engaging in supportive peri-implant maintenance regimen have a higher risk of peri-implantitis compared to those following a regular maintenance regimen [15]. A systematic review by Roccuzzo et al. (2018), based on 18 clinical studies concluded that regular supportive care following peri-implantitis therapy resulted in high implant survival in the medium-long term, improved clinical parameters and stable peri-implant bone levels in the vast majority of patients [16].

## 4. Peri-Implant Lesions Features

In principle many similarities exist in the immune response of tissues surrounding dental implants and those around teeth, against microbial biofilm challenge, and one may assume that peri-implant infections can be treated like periodontal ones, but relevant differences exist [17,18,19,20,21].

Carcuac and Berglundh (2014) when comparing human biopsies from 40 peri-implant lesions versus 40 periodontal lesions found that the former were larger, closer to alveolar bone, not surrounded by connective tissue and had a greater number of inflammatory cells [22].

In a study on experimental gingivitis and peri-implant mucositis in humans, Salvi et al. (2012) aimed to assess the host-derived factors mostly involved in the pathogenetic process [23]. They measured various biomarkers among which matrix metalloproteinase-8 (MMP-8) levels in gingival and peri-implant crevicular fluid [23]. MMP-8 (collagenase-2 or neutrophil collagenase) is a marker of polymorphonuclear leukocytes function, involved in the breakdown of collagen type I, a main component of connective tissues. MMP-8 level in gingival crevicular fluid and peri-implant sulcular fluid is strongly associated with inflammation and tissue degradation [24,25]. Salvi et al. reported that, regarding total MMP-8 concentration, implants reacted more severely than teeth to microbial biofilm challenge, but also recovered more slowly [23]. No significant differences were found for other biological markers (interleukin-1 beta and total microbial DNA counts) [23].

Such differences between periodontal and peri-implant infections involve the susceptibility to developing the disease (also related to different microbial composition of the biofilm and distinct features of host response [26], and the ability to successfully treat the affected tissues, as well as the maintenance of the treatment result.

## 5. Peri-Implant Treatment Effectiveness

Ghighi et al. (2018) reported that peri-implantitis and periodontal disease progressed by different mechanisms, and that periodontal tissues mostly responded to initial therapy while only a limited response was noted from peri-implant tissues [27]. The latter exhibited a distinct cytokine profile compared to non-responding periodontitis sites.

Relative to the stability or success of peri-implant treatment, Carcuac et al. (2020) in a prospective study on 73 patients demonstrated a 44% (57/130 implants) recurrence and progression of disease five years after surgical therapy [28]. In a study evaluating the clinical outcomes of supportive therapy following peri-implant surgical treatment of peri-implantitis in 24 patients (36 implants), Heitz-Mayfield et al. (2018) showed a recurrent breakdown at 58% of implants (37% of patients) treated five years earlier [29].

The literature in general shows limited and unpredictable success in treating the peri-implant lesions, with frequent esthetic, phonetic and food collection issues especially related to surgical peri-implantitis treatment [30,31,32,33].

## 6. Early Peri-Implantitis Risk Assessment

Based on the above literature analysis it seems evident that disease detection at the subclinical level is paramount to take measures for preventing major damage to peri-implant tissues, avoiding considerable discomfort to patients. In fact, advanced peri-implantitis often requires invasive and unpredictable treatment approaches [30].

In addition, according to the literature, peri-implant lesions may have systemic effects in the same way periodontal lesions do, as suggested by clinical and experimental studies [34,35]. Radaelli et al. (2021), in a review of the literature stated: “peri-implantitis has been shown to induce systemic changes at different levels, including blood cell count, serum biochemical parameters, and cytokine levels, which may have an influence on systemic conditions” [36]. The potential for systemic inflammation, including brain inflammation, related to peri-implantitis has been suggested by other recent reviews by Assery et al. (2023), and Tessarin et al. (2024), further underscoring the need for accurate and predictive diagnostic tools [37,38].

Early identification of a shift from healthy condition is difficult using conventional chairside diagnostic methods like probing of peri-implant pockets and visual inspection to identify signs of inflammation (bleeding, erythema, ulceration, and suppuration). Bleeding on probing (BOP) in particular has been shown not to be a reliable indicator of peri-implantitis [39,40,41]. Monje et al. (2021), Monje & Salvi (2024) and Yu et al. (2024) reviewed and discussed the poor accuracy of probing depths and BOP in early peri-implant disease detection, highlighting the considerable false positive rate associated with BOP [42,43,44]. These reviews emphasized the need for complementary diagnostic methods to monitor peri-implant conditions, especially two- or three-dimensional imaging for radiographic assessment of crestal bone levels [42]. However, these parameters require that bone and supporting tissues around implants must be lost prior to being able to formulate a diagnosis of peri-implantitis, a situation challenging to recover.

Several epidemiological studies and systematic reviews have established that peri-implantitis can be associated with many risk factors and risk indicators [45,46,47,48,49,50]. Some tools to assess the individual risk for peri-implantitis, considering multiple factors, have been proposed, like the Implant Disease Risk Assessment (IDRA) [4], and the Peri-implantitis Risk Assessment (PiRA) [5]. These tools are commendable as they identified and combined relevant patient-related and treatment-related factors, both modifiable and non-modifiable, each with a different impact on the incidence of peri-implantitis. Even if retrospective studies aiming at testing these tools (especially the IDRA) suggested they have a good accuracy in determining the risk of developing peri-implantitis [51,52], their use is rather time consuming and does not seem practical when a periodical re-assessment of the peri-implant tissue condition is intended. To this purpose, a protocol based on a simple, fast, and reliable point-of-care tool, possibly based on a single biomarker assessment, would be more appropriate and preferred by most clinicians.

## 7. Biomarkers Detection for Assessing Peri-Implant Tissue Inflammation

In the last years there has been growing attention to the role of tissue biomarkers as having a potential predictive value for assessing the status of peri-implant inflammation, thereby representing an additional assistance for early detection of an alteration from the physiological condition [53,54,55,56,57]. The most investigated biomarkers so far include pro-inflammatory cytokines (e.g., IL-1 beta, IL-6, tumor necrosis factor-alpha), anti-inflammatory cytokines (IL-2, IL-10, interferon gamma), chemokines (IL-8, macrophage inflammatory protein), osteoclastogenic-related factors (receptor activator of nuclear factor kappa B ligand (RANKL), and osteoprotegerin (OPG)), oxidative stress biomarkers (malondialdehyde), and enzymes (matrix metalloproteinases, Cathepsin K, Aspartate Aminotransferase, Alkaline phosphatase) [56].

Currently, the most promising and most studied is MMP-8. However, it must be clarified that different forms of MMP-8 exist in the tissue, and it is mandatory to distinguish among them. Atanasova et al. specifically addressed this issue, and explained how critical it is to differentiate total MMP-8 (tMMP-8), latent proMMP-8 and active MMP-8 (aMMP-8) in the oral health biomarker investigation, as they have distinct activities and importance [57,58,59]. In fact, latent proMMP-8 is enzymatically inactive (it has no collagenolytic action) [24,60,61]. Upon host or microbial proteases action, or when stimulated by reactive oxygen species, proMMP-8 is converted into the active form (aMMP-8), which can degrade various collagen types (type I and III), extracellular matrix proteins, complement factors, and further biomolecules [24,62]. aMMP-8 is the specific form of the enzyme that has been found to be involved in the pathogenesis of periodontal and peri-implant disease, producing irreversible tissue destruction [63]. Therefore, aMMP-8 is responsible for the enzymatic tissue breakdown at the active disease sites and progressive lesions in the supra-mentioned oral and systemic conditions, and its assessment and quantification can be critical to detecting the disease stage [24].

## 8. Active MMP-8 as a Reliable Indicator of Peri-Implant Disease

Given the above considerations, aMMP-8 appears to be among the most useful indicators for identifying the early and often invisible alteration of the supporting structures of implants before the tissue breakdown becomes clinically visible by increasing probing depths and radiographic bone loss [64]. Therefore, assessment of aMMP-8 levels can help in early disease detection and represents a valuable complement in the diagnostic assessment of peri-implantitis.

A point-of-care (PoC)/chairside test to identify and quantify the presence of aMMP-8 in the peri-implant sulcular fluid (ImplantSafe, Dentognostics GmBH, Solingen, Germany) has been introduced in mid 2010s’ as an adjunctive tool in the dental office for the simple and fast assessment of peri-implant tissue status [65,66]. It consists of a site-specific lateral flow immunotest based on monoclonal antibodies technology, able to specifically detect aMMP-8 in PISF, and derives from an analogue PoC test earlier developed for periodontal disease (PerioSafe, Dentognostics GmBH). PISF is collected by a standard technique using paper strips which are then quantitated in a digital reader (ORALyser, Dentognostics GmBH) in 5 min. This quantitative PoC test has been validated independently in different Countries (Finland, Germany, Italy, Nigeria, Turkey, the Netherlands, and the United States) [67,68,69,70,71,72,73]. In these studies, the test demonstrated a diagnostic sensitivity and specificity of 76–83% and 96%, respectively.

Since the introduction of ImplantSafe, many prospective and retrospective clinical studies [66,74,75,76,77,78,79,80,81,82,83,84,85,86,87,88,89], as well as systematic reviews [53,54,56,90,91,92] have demonstrated the usefulness of aMMP-8 assessment in the early diagnosis of peri-implant inflammation. A recent systematic review by Del Fabbro et al. (2026), conducted in accordance with Preferred Reporting of Systematic Review and Meta-Analysis of diagnostic test accuracy studies (PRISMA-DTA), specifically addressed the diagnostic potential of aMMP-8 PoC immunoassay for peri-implant disease [93]. In that review only comparative clinical studies published between 2016 and 2025 were included. The literature search was conducted on Pubmed, Embase and Scopus databases, supplemented by manual search and grey literature search. The details of the search strategy, inclusion and exclusion criteria, selection process, data extraction, and study quality assessment are fully described in that paper [93]. The main features of the eight studies included in that systematic review are summarized in Table 1. These studies reported measures of diagnostic accuracy like specificity, sensitivity, accuracy, area under the ROC (Receiver Operating Characteristic) curve, and correlation with clinical parameters (bleeding on probing, probing depth, clinical attachment loss, radiographic bone loss). Based on the diagnostic accuracy measurements, the studies proposed predictive cutoff thresholds for aMMP-8 concentration, ranging from 15.3 ng/mL [77,78] to 33.7 ng/mL [81,82], to differentiate between healthy and diseased peri-implant tissues, and between peri-implant mucositis and peri-implantitis. Based on the results of these studies, Del Fabbro et al. (2026) globally concluded that aMMP-8 levels were consistently more elevated in peri-implantitis compared to mucositis and healthy sites, with very good to excellent values of diagnostic measures of accuracy [93]. A study by Guarnieri et al. (2024) compared the post-treatment aMMP-8 levels in gingival crevicular fluid and PISF before and after non-surgical treatment of periodontitis and peri-implantitis, respectively [76]. From the above findings, it can be concluded that aMMP-8 in PISF represents a predictable biomarker with high diagnostic accuracy for early detection of collagen degradation process, and that standardization of cutoff thresholds as well as validation through longitudinal studies are required before integrating the tool in routine clinical practice.

The above-described studies overall represent a body of evidence suggesting that the clinical use of such PoC/chairside test can be valuable not only for screening and prevention of peri-implant disease onset, but also to predict future progression or recurrence, to monitor treatment response and, importantly, to determine the appropriate maintenance regimen.

From a methodological standpoint, according to the above-cited systematic review the studies described in Table 1 have an overall high quality, all having been ranked 7/9 or more according to the Newcastle-Ottawa Scale, except the Guarnieri et al. (2022) study [77,78] which was ranked 6/9 in the systematic review by Del Fabbro et al. (2026) [93]. Nevertheless, the diagnostic accuracy estimates in Table 1 should be interpreted with caution due to the heterogeneity in aims, clinical protocols, patient selection, index test blinding, and reference standard inconsistency across these studies.

## 9. From Periodontal to Peri-Implant Maintenance

Regular maintenance through periodical sessions of professional oral hygiene is recognized as an essential procedure to preserve dentition health and support, and to avoid further progression of periodontal tissue breakdown after periodontitis treatment [94,95,96].

Regarding the proposed recall frequency for periodontal maintenance after completion of periodontal therapy there is wide heterogeneity in the published literature, with intervals ranging between two weeks and longer than 12 months. However, as underlined in a review by Trombelli et al. (2020), there is no consistent evidence regarding the effectiveness of a specific interval for supportive periodontal maintenance [96]. In general, available studies favor the concept that shorter recall intervals for supportive periodontal therapy are related to lower tooth loss rate, but the optimal frequency is unclear, while it seems clear that intervals should be tailored to each individual case [95].

It has been proposed that the frequency of periodontal recalls should be determined through patient-based risk assessment tools used after completion of active periodontal therapy, and that in addition to severity of the disease, one critical index to adjust the intervals is bleeding on probing score [97]. The importance of BOP in scheduling recall frequency was also highlighted by Trombelli et al. in a cohort of 109 patients undergoing supportive periodontal therapy and followed for 5.6 ± 2.2 years (range: 3.7–15.6 years) [96,98]. However, as underlined in previous sections, bleeding on probing is unreliable when peri-implant tissue condition must be determined. Furthermore, such index should be considered cautiously in smokers, given the known vasoconstriction effect caused by smoking.

Trombelli et al. underlined that strong evidence to guide the selection of an appropriate recall frequency of periodontal supportive therapy based on individual periodontal risk assessment is still lacking. Indeed, tools for evaluating such risk have been proposed for long [99,100], and it is advised that patients with a similar risk score be included in a supportive periodontal therapy protocol having a similar recall frequency [96]. Previous studies by Matuliene et al. (2010), and Trombelli et al. (2017) indicated that patients with higher periodontal risk scores tend to have a greater tooth loss rate than low-risk patients, suggesting the optimal recall intervals for the former should be shorter than for the latter [98,101]. These considerations highlight the need for personalized primary and secondary prevention regimens in patients having different risk profiles to decide the appropriate interval for supportive periodontal therapy. The same concepts may apply to the implant patient: in fact, like for teeth, regular implant maintenance is of paramount importance to preserve peri-implant tissue health. A survey published in 2018 aiming at defining the most appropriate recall regimen and professional maintenance care protocol for both periodontal and implant patients, based on a consensus of expert practitioners with at least 20 years of experience, concluded that such protocol must be individually determined, and a baseline condition identified [95]. In this survey the experts agreed that the recall frequency must have a specific periodicity, depending on the tissue health/disease condition, and bone levels should be radiographically controlled at least every two years or shorter in case of specific needs [95].

## 10. Biomarker Guided Implant Maintenance

Peri implant maintenance involves normal recare procedures that are similar to those used on teeth. However, special instruments are necessary because of the titanium surface of the implant and implant abutment, which can easily be scratched and roughened by the harder standard dental curettes. Therefore, special instruments, including coated and plastic scalers are recommended.

Given the greater susceptibility of peri-implant tissues to develop biofilm-related inflammatory disease, as compared to periodontal tissues, it may be hypothesized that the recall intervals for implant patients should be at least similar or even shorter than periodontal patients. The above cited panel of experts unanimously agreed that for an implant maintenance program the appropriate timing should be identical to those adopted for periodontally compromised patients. The panel generally advised intervals of six months between visits for low-risk implant patients (those who demonstrate no susceptibility to inflammatory disease) and 3–4 months otherwise [95]. A systematic review by Monje et al. (2016) underlined the importance of a regular peri-implant maintenance therapy, recognized it must be tailored to the patient’s risk profiling, and suggested a minimum recall interval of 5 to 6 months, even for low-risk patients with healthy peri-implant tissues [102].

Since the risk of developing peri-implantitis varies among subjects as it depends on individual factors, also preventive measures (in terms of recall frequency for maintenance care) should be targeted to individual needs of each patient, as was discussed above for periodontal patients. Given the dynamic nature of the disease, and the possible changes in all factors that may alter tissue condition and disease progression susceptibility, periodical assessment of risk stratification are required.

The possibility to determine the actual health or diseased status of peri-implant tissues with the aid of a simple and fast chairside test, might be extremely helpful for clinicians to determine the actual needs of the patients in terms of maintenance frequency and specific protocol, establishing individual maintenance regimens. To this regard, the PoC aMMP-8 immunotest can provide useful information to help clinicians in categorizing the implant patients and decide the optimal recall scheduling. The study by Lähteenmäki (2022) based on aMMP-8 concentration values in PISF as related to peri-implant tissue condition suggested to consider three categories: low risk (aMMP-8 level < 20 ng/mL), moderate risk (aMMP-8 level between 20 and 80 ng/mL) and high risk (aMMP-8 level > 80 ng/mL) [79]. The diagnostic accuracy measures of that study, based on the above thresholds, were extremely high. These proposed thresholds deriving from real-time diagnostic chairside immunotest, could represent a basis (to be further confirmed with longitudinal studies), to set personalized implant maintenance regimen.

Based on the considerations discussed throughout this paper, the authors are proposing the Biomarker-Guided Implant Maintenance (BGIM) protocol, schematically presented in Table 2.

The advantage of such proposed protocol is that through a simple and fast procedure the patients can be categorized based on individualized peri-implant inflammation risk in one of three maintenance protocols with different recall frequency.

From a practical perspective, the implementation of the BGIM protocol requires the availability of a point-of-care test capable of quantifying aMMP-8 levels in peri-implant sulcular fluid together with a digital reader for result interpretation. The sampling procedure requires only a few minutes and can be easily integrated into routine maintenance visits. Although the cost of chairside testing may vary across countries and clinical settings, it is expected to be modest compared with the biological and economic burden associated with the treatment of advanced peri-implantitis. The aMMP-8 cutoff values proposed in the BGIM protocol represent a pragmatic synthesis based on currently available evidence and require prospective clinical validation before adoption in routine practice.

## 11. Scientific Rationale for BGIM Parameters

- aMMP-8: Selected as the primary biological parameter because it is the only currently available real-time, chairside-quantifiable biomarker with validated diagnostic accuracy for active peri-implant collagenolysis, capable of detecting subclinical inflammation before clinical signs emerge [63,79].
- Oral hygiene status: Biofilm accumulation is the primary etiological factor of peri-implantitis [2,3]. Poor oral hygiene is consistently associated with higher peri-implant disease risk across epidemiological studies [46,48].
- History of periodontitis: Patients with a history of periodontitis exhibit a significantly elevated susceptibility to peri-implantitis, likely due to shared genetic, immunological, and microbiological risk profiles [40,49].
- Smoking: Smoking impairs immune response, reduces vascular perfusion, and masks clinical inflammation signs (including BOP), making it an independent risk factor for peri-implantitis and a confounder of clinical monitoring [46,48].
- Systemic diseases: Conditions such as uncontrolled diabetes mellitus compromise immune response and tissue healing, increasing susceptibility to peri-implant inflammation [48,49].
- Prosthetic design: Prosthetic design that hinders access for oral hygiene procedures directly contributes to biofilm accumulation and is a known implant-related risk factor [50].
- Patient dexterity: Compromised dexterity limits the effectiveness of homecare, independently elevating disease risk and necessitating more frequent professional maintenance.

**Figure 1:**
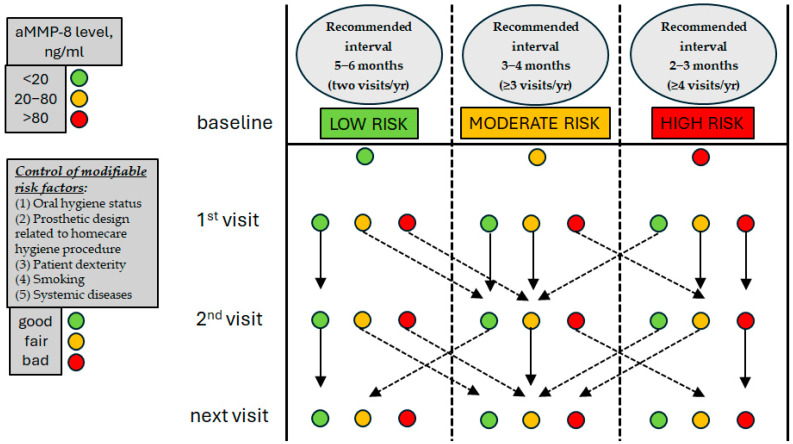
Clinical decision flowchart for BGIM. aMMP-8 should be first tested at crown delivery (baseline) and then the PoC test should be repeated at every hygiene recall along with risk factors re-evaluation and patients’ re-motivation. The recommended interval should be adjusted according to aMMP-8 levels and assessment of other factors. Patients may change their category along with follow-up if the aMMP-8 level changes (dotted arrows). If the test gives a worse result the patient is immediately placed in a stricter maintenance regimen. In patients initially at high risk, if the aMMP-8 level improves markedly from >80 to <20 ng/mL, the patient is immediately placed in the moderate risk category. If the improvement is slight, the patient is moved to a less strict category only after confirmation at the subsequent visit. Solid arrows indicate the patient remains in the same risk category.

The rationale for combining these factors into a single risk category follows the principle that peri-implantitis is multifactorial, and that cumulative risk—even when driven by a single dominant factor—should determine the most protective maintenance interval. The Authors suggest that a patient should be put into the appropriate recall frequency category based on the highest risk score. Even if only one factor is rated at high risk, the patient should be placed in the most stringent maintenance category. In Figure 1 a flowchart is schematically depicted showing how a clinician can utilize the BGIM protocol step-by-step, from baseline to subsequent visits. The figure shows that the risk of disease progression is a dynamic concept: it may change if some risk factor changes over time, and to preserve peri-implant tissue health, maintenance scheduling must be adapted and interpreted as a dynamic concept as well. If the risk increases the patient can be placed immediately on a stricter regimen, and if the risk decreases (improvement of patient dexterity, quit smoking, improved systemic disease control), and a decreased aMMP-8 level is detected, the patient may be moved to a less strict maintenance regimen.

No clinical index with unreliable correlation with peri-implant tissue condition (like probing depth and bleeding on probing) is considered. Furthermore, one should consider that the susceptibility of patients might change over time due to several confounders, change in lifestyle habits and general health issues, and to a possible underestimation of the actual risk, leading to inappropriate maintenance schedule. If during the follow-up a worsening of the peri-implant tissue status is detected, reflected by an increase of the aMMP-8 level, the patient can be promptly shifted to a higher risk category with stricter intervals before progressive loss of peri-implant support occurs.

The BGIM tool is still hypothetical and should be validated in terms of both accuracy and clinical benefit, by multicenter prospective studies in which patients having dental implants, with different levels of risk at baseline/first visit are assessed, categorized, placed under the appropriate maintenance regimen, and followed for at least 3–5 years. In these studies, it would be interesting to compare the BGIM protocol with other multifactorial peri-implant risk assessment tools currently available like the IDRA [4] or the PiRA [5], to evaluate the agreement in categorizing the patients.

BGIM protocol may be prone to false positives/negatives: including the risk of over-recall in low-risk patients and, more critically, under-recall in patients with subclinical peri-implant activity. We note that the BGIM is a cautious protocol (i.e., more frequent recall when any risk factor is elevated), precisely to minimize false negative consequences.

One of the limitations of BGIM protocol is that the aMMP-8 cutoff thresholds proposed (<20 ng/mL, 20–80 ng/mL, >80 ng/mL) are derived primarily from a single study (Lähteenmäki et al., 2022 [79]), while the literature reports a range of proposed thresholds (15.3–33.7 ng/mL) for distinguishing healthy from diseased peri-implant tissues. This variability may be affected by differences in patient populations, sampling techniques, disease severity definitions, and assay conditions. Differences in implant systems, prosthetic design, maintenance setting may also contribute to variability across studies. Until these thresholds are confirmed through large multicenter longitudinal studies, clinicians should interpret aMMP-8 levels as part of a holistic risk assessment rather than as absolute decision boundaries. The thresholds for aMMP-8 proposed in BGIM of course are not inalterable but might be changed if future validation studies will provide evidence of more reliable values to discriminate between different peri-implant tissue conditions.

Another limitation is that the current BGIM framework relies primarily on a single biomarker. Although aMMP-8 currently represents the most validated and clinically accessible biomarker for peri-implant inflammation, future diagnostic approaches may benefit from multiple biomarker panels including inflammatory cytokines, osteoclastogenic markers, or oxidative stress indicators [54,56]. The BGIM framework is intended to remain adaptable to incorporate such advances as chairside diagnostic technologies evolve.

## 12. Conclusions

Based on the narrative review and proposed protocol, the following key conclusions can be drawn:

- **Peri-implantitis: A Persistent Challenge:** Peri-implantitis remains a significant biological complication in dental implantology, with variable prevalence and often unpredictable treatment outcomes. Early detection is crucial to prevent extensive damage and discomfort to patients.
- **Limitations of Conventional Diagnostics:** Traditional diagnostic methods, such as probing depths and bleeding on probing (BOP), have shown poor accuracy in early peri-implant disease detection, often indicating disease only after significant bone and tissue loss has occurred.
- **aMMP-8 as a Reliable Biomarker:** Active Matrix Metalloproteinase-8 (aMMP-8) has emerged as a highly promising and reliable biomarker for identifying early and active peri-implant inflammation. Its presence is strongly associated with tissue degradation at active disease sites.
- **Point-of-Care (PoC) Testing for aMMP-8:** The availability of rapid, chairside point-of-care tests for aMMP-8 in peri-implant sulcular fluid offers a practical and efficient tool for clinicians to assess peri-implant tissue status in real-time.
- **Biomarker-Guided Implant Maintenance (BGIM) Protocol:** The proposed BGIM protocol integrates aMMP-8 levels, along with other clinical and patient-related factors (oral hygiene, history of periodontitis, smoking, systemic diseases, prosthetic design, and patient dexterity), to provide a comprehensive and individualized risk assessment for peri-implant inflammation.
- **Personalized Maintenance Scheduling:** The BGIM protocol may enable personalized implant maintenance scheduling, recommending increased recall frequency for patients with higher risk of peri-implantitis. This individualized approach aims to prevent disease progression and enhance implant longevity.
- **Improved Clinical Outcomes:** By facilitating early intervention and tailored maintenance, the BGIM protocol has the potential to improve clinical outcomes, reduce the incidence of advanced peri-implantitis, and ultimately enhance the success of dental implant treatment. However, BGIM protocol should be framed as a promising but preliminary tool, not as an established clinical standard, and its effectiveness in improving long-term implant outcomes requires confirmation in longitudinal clinical studies.

## Figures and Tables

**Table 1:** Main characteristics of the studies investigating the diagnostic value of aMMP-8 for peri-implant disease.

First Author, Year	Study Objective	Sample Size/Follow-Up	Outcomes Assessed	Key Statistical Results/Diagnostic Accuracy Measures	Conclusions
Fragkioudakis et al., 2025 [80]	To evaluate the diagnostic sensitivity and specificity of aMMP-8	Healthy/mucositis group (*n* = 54 patients); PI group (*n* = 48 patients)	Clinical parameters: BOP, PD, REC, and CAL. aMMP-8 levels, Diagnostic performance metrics for cutoff 20 to 30 ng/mL	Significantly higher BOP, PD, CAL, aMMP-8 levels in PI group. Se: 81.3%, Sp: 74.1%, ROC-AUC: 0.80	aMMP-8 is a promising biomarker for PI, showing elevated levels in affected patients.
Guarnieri et al., 2024 [76]	To relate aMMP-8 in PISF/GCF with clinical indices pre-/post-treatment.	45 implants/45 patients; Healthy (*n* = 15)/Peri-implant mucositis (*n* = 15)/PI (*n* = 15) 3 months follow-up	aMMP-8 levels, PD, MBL	PISF aMMP-8 remained stable after therapy. GCF levels reduced significantly (*p* < 0.01).	aMMP-8 is a stable marker of peri-implant disease state.
Xanthopoulou et al. 2024; [81] Xanthopoulou et al., 2023 [82]	To validate aMMP-8 and azurocidin as biomarkers for identifying peri-implant diseases.	80 implants/80 patients; Healthy peri-implant mucosa (*n* = 27)/Peri-implant mucositis (*n* = 41)/PI (*n* = 12)	Correlation aMMP-8 levels with clinical indices PD, CAL, BOP and PLI; ROC-AUC analysis for sensitivity/specificity	Increasing probing depths of the sampled site and aMMP-8 levels were significantly correlated. Cutoff: 33.7 ng/mL, Se: 91%, Sp: 75%, ROC-AUC: 0.80, accuracy: 77.5%	aMMP-8 is accurate and reliable for PI disease diagnosis.
Guarnieri et al., 2022 [77], Guarnieri et al., 2022 [78]	To evaluate MBL progression and aMMP-8 as predictors of PI	80 implants/80 patients (24 PI cases) 5-year follow-up	aMMP-8 levels, marginal bone loss	aMMP-8 > 15.3 ng/mL at 6 months predicts the onset of PI at 5 years Cutoff: 15.3 ng/mL, Se: 95.8%, Sp: 100%	No correlation between early bone loss nor BOP and PI onset; aMMP-8 > 15.3 ng/mL at baseline and 6-month is related to high risk of PI development and greater bone loss at 2 and 5 years.
Lähteenmäki et al., 2022 [79]	To assess aMMP-8 PoC test for PI diagnosis.	68 implants/68 patients; Healthy (*n* = 42), PI (*n* = 26)	aMMP-8 levels, clinical indices: PD, BOP and MBL	aMMP-8 PoC (adjusted model); correlates with clinical severity. Cutoff: 20 ng/mL, Se: 75%, Sp: 84.2%, ROC-AUC: 0.88, accuracy: 80.6%	Quantitative aMMP-8 excels as a real-time diagnostic tool to detect active collagenolysis affecting peri-implant tissues (≥80 ng/mL: rapid progression)
Lähteenmäki et al., 2020 [83]	To assess aMMP-8 PoC test compared to IFMA, for PI diagnosis.	52 implants/50 patients; Healthy (*n* = 26), PI (*n* = 26)	aMMP-8 (PoC), aMMP-8 (IFMA), MMP-8/TIMP-1, PMN elastase, MPO, TIMP-1, active-MMP-9, pro-MMP-9 and BOP	aMMP-8 outperforms other markers for PI detection. Cutoff: 20 ng/mL, Se: 100%, Sp: 100%, ROC-AUC: 1, accuracy: 100%	aMMP-8 is highly reliable for PI diagnosis.
Lupi et al., 2019 [74]	To evaluate chairside aMMP-8 test for differentiation between healthy and diseased peri-implant tissue	50 implants/50 patients 1 to 7 years	aMMP-8 levels, sensitivity, accuracy vs. clinical indices (BOP, PD, PLI, F (peri-implant inflammation index))	MMP-8 correlates with inflammation indices. No significant difference between the two PISF sampling methods. Cutoff: 25 ng/mL, Se: 70%, Sp: 80%, accuracy: 72%	Chairside aMMP-8 test showed a positive, but not high, correlation between the test results and peri-implant indexes
Thierbach et al., 2016 [84]	To analyze aMMP-8 levels in PISF in smokers vs. non-smokers with PI at baseline and 6 months after treatment	29 patients with PI (11 periodontally healthy, 18 periodontitis) 6 months	aMMP-8 levels pre- and post-treatment; PD, CAL, BOP, IL-1 risk type	aMMP-8 levels correlated with GI, PD, and is higher in periodontitis than healthy/gingivitis patients (*p* < 0.05). aMMP-8 decreases after treatment in PI sites (*p* < 0.05) in both smokers and non-smokers	aMMP-8 reflects inflammation, its levels are higher in peri-implantitis and decrease by treatment.

MMP: matrix metalloproteinase; aMMP: active matrix metalloproteinase; MPO: myeloperoxydase; IL: interleukin; PI: peri-implantitis; PISF: peri-implant sulcular fluid; GCF: gingival crevicular fluid; IFMA: immunofluorimetric assay; PoC: Point-of-Care; MBL: marginal bone loss; BOP: bleeding on probing; PD: probing depth; REC: recession; CAL: clinical attachment level; GI: gingival index; PLI: plaque index; F: peri-implant inflammation index; Se: sensitivity; Sp: specificity; ROC-AUC: Areas Under the Receiver Operating Characteristic Curve.

**Table 2:** Proposal for a biomarker-guided implant maintenance program based on peri-implant risk of inflammation.

Risk of Peri-Implant Inflammation		Healthy/Low Risk	Moderate Risk	High Risk
Yearly recall frequency (recommended interval)		Two visits (5–6 months)	at least 3 visits (3–4 months)	4 or more visits (2–3 months)
Factors to be considered	aMMP-8 level	<20 ng/mL	20–80 ng/mL	>80 ng/mL
	Oral hygiene status	Good	Average	Poor
	History of periodontitis	No	Yes	Yes
	Smoking	No	Past smoker	Yes
	Systemic diseases	No	No/good control	Yes
	Prosthetic design related to homecare hygiene procedures	Easy access	Limited access	Difficult access
	Patient dexterity assessment	Good	Limited	Compromised

## Data Availability

No new data were generated in this study. Data sharing is not applicable to this article.
